# Phenotypic selection during laboratory evolution of yeast populations leads to a genome-wide sustainable chromatin compaction shift

**DOI:** 10.3389/fmicb.2022.974055

**Published:** 2022-10-13

**Authors:** David F. Moreno, Murat Acar

**Affiliations:** ^1^Department of Molecular Cellular and Developmental Biology, Yale University, New Haven, CT, United States; ^2^Systems Biology Institute, Yale University, West Haven, CT, United States; ^3^Department of Medical Biology, School of Medicine, Koc University, Istanbul, Turkey

**Keywords:** inheritance, epigenetic mechanisms, microbial evolution, memory, Lamarckian, ATAC-seq, chromatin accessibility

## Abstract

In a previous study, we have shown how microbial evolution has resulted in a persistent reduction in expression after repeatedly selecting for the lowest P_GAL1_-YFP-expressing cells. Applying the ATAC-seq assay on samples collected from this 28-day evolution experiment, here we show how genome-wide chromatin compaction changes during evolution under selection pressure. We found that the chromatin compaction was altered not only on GAL network genes directly impacted by the selection pressure, showing an example of selection-induced non-genetic memory, but also at the whole-genome level. The GAL network genes experienced chromatin compaction accompanying the reduction in P_GAL1_-YFP reporter expression. Strikingly, the fraction of global genes with differentially compacted chromatin states accounted for about a quarter of the total genome. Moreover, some of the ATAC-seq peaks followed well-defined temporal dynamics. Comparing peak intensity changes on consecutive days, we found most of the differential compaction to occur between days 0 and 3 when the selection pressure was first applied, and between days 7 and 10 when the pressure was lifted. Among the gene sets enriched for the differential compaction events, some had increased chromatin availability once selection pressure was applied and decreased availability after the pressure was lifted (or vice versa). These results intriguingly show that, despite the lack of targeted selection, transcriptional availability of a large fraction of the genome changes in a very diverse manner during evolution, and these changes can occur in a relatively short number of generations.

## Introduction

Epigenetics is a gene-expression control mechanism that can help organismal adaptation to the environment. Built on epigenetic mechanisms, Lamarckian theory of evolution has received a significant amount of attention in the past two decades (Day and Bonduriansky, 2011; Burggren, 2014; Skinner et al., 2015). In the framework of Lamarckian evolution, environment directly alters phenotype throughout generations (Skinner, 2015), and epigenetic mechanisms can act as the facilitator for the environment to change phenotypic variation and its inheritance (Skinner, 2015).

Epigenetic, or more broadly, nongenetic inheritance can be mediated in several ways, including through the inheritance of epigenetic states, cytoplasmic factors, and nutrients. In this context, nongenetic inheritance could be advantageous over genetic inheritance by circumventing its limitations through the decoupling of phenotypic change from the genotype (Bonduriansky and Day, 2009). The acquired inheritance is a long-term consequence of epigenetic memory, defined as the stable propagation of a change in gene expression potentially induced by environmental stimuli (D’Urso and Brickner, 2014). The importance of epigenetic memory has been shown at the single-cell level in cancer progression (Meir et al., 2020) and bacterial virulence (Ronin et al., 2017). Moreover, its dynamics has been modeled (Bintu et al., 2016) and their action mechanism on pluricellular organisms has been extensively reviewed (Migicovsky and Kovalchuk, 2011; Iwasaki and Paszkowski, 2014).

Several studies have focused on nongenetic inheritance across generations (Acar et al., 2005, 2008; Tyedmers et al., 2008; Huang, 2009; Halfmann et al., 2012; Peng et al., 2015; Bódi et al., 2017; Chatterjee and Acar, 2018; Xue and Acar, 2018a,b; Stajic et al., 2019). For example, phenotypic heterogeneity as an evolvable trait facilitates adaptive evolution (Bódi et al., 2017). In another study, epigenetic gene silencing has been shown to change the mechanism and rate of evolutionary adaptation (Stajic et al., 2019). Finally, in a gene network setting, work from our laboratory provided an example for the role played by epigenetic mechanisms on evolution (Luo et al., 2020). We showed the role played by epigenetic inheritance in microevolution. Subjecting yeast cells to repeated environmental selection based on the P_GAL1_-YFP expression (Acar et al., 2005, 2008, 2010; Elison et al., 2018; Luo et al., 2018) over a period of 7 days, we observed gene expression reductions for the samples sorted for the lowest YFP expression; the expression reductions persisted even after the selection pressure was lifted during an additional 21 days period. Epigenetic and genetic factors were found to contribute to these persistent expression level reductions (Luo et al., 2020).

**Figure 1:**
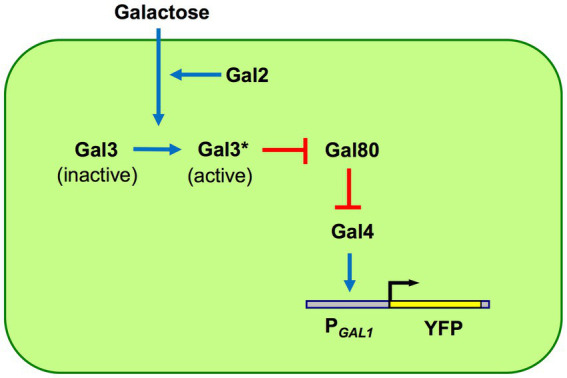
GAL network components and architecture. The network is built by four regulatory proteins: Gal2 transporter, Gal3 inducer, Gal4 activator, and Gal80 repressor. The galactose-bound active state of Gal3 is denoted by Gal3*. The GAL1 promoter (P_GAL1_) has binding sites for Gal4 activators; unrepressed Gal4 activates the P_GAL1_ as well as other network promoters, except the GAL4 promoter which is constitutively active. Pointed blue arrows reflect activation while blunt red arrows reflect inhibition. Network activity is reported using a P_GAL1_-YFP reporter.

To track evolution at the gene network level, using networks with well-characterized components is a practical necessity for faithful interpretation of results as phenotypic evolution itself and its root causes are already often challenging to sort out. The GAL network in yeast is arguably the most suitable small-scale transcriptional gene network to use as an experimental evolution model. There is more than half a century of research on the genetics and biochemistry of this network, having characterized its regulatory and enzymatic components and their interaction in the network. The activity of the GAL is often reported by the P*_GAL1_*-YFP construct integrated into the yeast genome. Galactose is taken up by the Gal2 proteins and other hexose transporters. The inducer Gal3 is activated by galactose and active Gal3 proteins bind to the Gal80 repressor. When Gal80 repressors are bound by active Gal3 inducers, Gal4 activators are no longer repressed by Gal80, and therefore, they can turn on transcription from the *GAL1* promoter (Figure 1).

ATAC-seq, transposase-accessible chromatin using sequencing, is an *in vitro* epigenome analysis assay that uses transposition of sequencing adaptors into native chromatin (Buenrostro et al., 2013). ATAC-seq captures open chromatin sites, elucidating single-nucleotide-resolution information about chromatin compaction. Since its original development, ATAC-seq has been applied to a variety of eukaryotic cell types or organisms, leading to the characterization of many different cellular processes and phenotypes as a function of chromatin accessibility (Friman et al., 2019; Kakebeen et al., 2020; Yang et al., 2020; Van Rechem et al., 2021). For example, an improved ATAC-seq method was applied to age-enriched yeast cells, which led to the discovery that global nucleosome occupancy did not significantly change with cellular age (Hendrickson et al., 2018b). Recent work from the Shapiro Lab has adapted the ATAC-seq method in a prokaryote (Bac-ATAC), showing that this technique is able to capture chromosome accessibility and compaction even in the absence of fully-fledged chromatin (Melfi et al., 2021).

Using ATAC-seq applied on samples collected from an evolution experiment, here we present how genome-wide chromatin compaction dynamically changed throughout evolution. We examined chromatin compaction both on the GAL network genes that were subjected to selection pressure and the rest of the yeast genome. We analyzed ATAC-seq peaks for temporal dynamics during evolution. Results from this study suggest that, in response to environmental pressures, eukaryotic genomes are much more plastic and interconnected than previously thought.

## Materials and methods

### Strain description, experimental setup, and culture growth conditions

One set of the microevolution experiments performed in our previous work (Luo et al., 2020) was done using the strain WP35 which was a haploid wild-type yeast strain carrying a single copy of the P_GAL1_-YFP reporter in the ho locus; this strain had a MATα W303 genetic background. The initial (pre-evolution) genotype of the strain used in our current study is the following:

*MATα, leu2-3,112, trp1-1, can1-100, ura3-1, ade2-1, his3-11,15, ho*::HIS5-P*_GAL1_*-YFP.

During those multi-day microevolution experiments, cell populations corresponding to each independent replicate were periodically collected/preserved as frozen stocks. For the current study, we have revived the L3 replicate (L for Low YFP; Luo et al., 2020) by streaking its cells on synthetic complete media plates containing 2% glucose, followed by plate growth for 48 h. Liquid cultures inoculated with those revived cells were grown at 30°C in a shaking-incubator (225 rpm) in a volume of 10 ml, in synthetic complete media containing 0.1% mannose as a non-inducing sole carbon source and grown for 22 h. After this initial liquid growth period, GAL1 expression was induced by transferring the cells to 10 ml fresh synthetic complete media containing 0.1% mannose and 0.2% galactose as carbon sources and grown in the shaker-incubator for another 24 h.

### Flow cytometry data acquisition

After the induction period described in the above section, the YFP expression distribution of 10,000 cells was measured by flow cytometry (FACSVerse, BD), using the FITC-A filter set. To minimize potential variations in size and/or morphology of the measured cells, we gated them by applying a narrow FCS-SSC range corresponding to the densest ~25% of the total cell population. We note that we did not perform any sorting experiments for the current study; cells revived from the frozen cultures (frozen on specific days of the evolution experiment for a specific experimental replicate) of our previous study (Luo et al., 2020) were analyzed by flow cytometry based on the above description.

### ATAC-seq sample preparation and obtaining the raw data

After the induction period described in the above section, yeast cells were prepared for ATAC-seq as described previously (Hendrickson et al., 2018a). Briefly, 5 million cells were pelleted (2 min/4600 rpm) and washed twice with Spheroplasting Buffer (SB: 1 M sorbitol, 40 mm HEPES [pH7.5], 10 mm MgCl_2_) at room temperature, and resuspended in 190 μl of SB + 10 μl of Zymolyase-100 T 10 mg/ml dissolved in SB. Cells were incubated at 30°C for 30 min with rocking. Spheroplasts were pelleted (2 min/4600 rpm), washed twice with SB at room temperature, and resuspended in 50 μl of Tagmentation Mix (25 μl Tagment DNA Buffer, 22.5 μl nuclease-free water, 2.5 μl Tagment DNA Enzyme1, Illumina #20034210). The tagmentation reaction was incubated at 37°C for 30 min with no shaking or mixing. Later, DNA was then purified with the QIAquick PCR Purification Kit (Qiagen #28104) following the manufacturer’s protocol, eluted in 11 μl of water, and stored at-20°C until ready for PCR. PCR reactions were set up as follows: we mixed 25 μl of NEBNext Hi-Fidelity 2X PCR Master Mix, 7.5 μl of water, 6.25 μl of universal primer (Ad1, 10 μm), 6.25 μl of barcoded reverse primer (Ad2.1-Ad2.12, 10 μm), and 5 μl of DNA from the previous step. The primers were HPLC-purified after synthesis; their sequences are listed on Supplementary Table S1. The thermocycler was programmed as follows: 72°C for 5 min (one cycle); 98°C for 30 s (one cycle); 98°C for 10 s, 63°C for 30 s; 72°C for 1 min (ten cycles); and hold at 4°C. The amplified libraries were cleaned up using Ampure XP magnetic beads (Beckman Coulter #A63880), first removing large amplicons (>1,000 bp) using 0.4× volume of beads, and later selecting fragments >100 bp using 1.5× volume of beads. The latter beads were washed twice with 80% ethanol, dried, and resuspended in 22.5 μl of water to elute the DNA. The final solution was repurified with the QIAquick PCR Purification Kit, eluted in 22 μl of water, and QC-analyzed on a 2100 BioAnalyzer Instrument (Agilent #G2939B).

After the QC, the libraries were pooled at an equimolar ratio for multiplexing and sequenced with a NovaSeq 6000 System (Illumina), generating 15 M of 100 bp paired-end reads per sample (Yale Center for Genome Analysis, West Haven). The reads were barcode-demultiplexed before further analysis.

### ATAC-seq data processing

Low-quality reads were removed, and adaptor contamination was trimmed using Trim Galore (v0.5.0; Krueger et al., 2018). Trimmed reads were mapped to the *Saccharomyces cerevisiae* genome assembly R64 (sacCer3) using Bowtie2 (v2.2.9; Langmead and Salzberg, 2012). Peaks were called using MACS2 (v2.1.1; Feng et al., 2012) and annotated using the HOMER program annotatePeaks.pl. (v4.10.3; Heinz et al., 2010). To identify the differentially open chromatin regions between specific time points, we first pooled the peaks from the two timepoints involved by merging the overlapping peaks using BEDTools (v2.30.0; Quinlan and Hall, 2010), then counting the reads located in the merged peaks using featureCounts (v1.6.3; Liao et al., 2014), and finally identifying the differentially binding regions using DESeq2 (v1.30.1; Love et al., 2014).

### Gaussian process test for dynamic time-course data and clustering

Standardized ATAC-seq counts for each peak (identified by MACS2) were fitted to a Gaussian process regression model (Yang et al., 2016) with a radial basis function (RBF) kernel plus a white noise kernel to represent a dynamic model, and a pure white noise kernel to represent the static model, respectively. A stringent *χ*^2^ test with one degree of freedom was applied to the log-likelihood ratio (LR) statistics, with $\mathrm{LR}=-2\ln\left({\hat{L}}_{RBF}-{\hat{L}}_{STATIC}\right),$ where ${\hat{L}}_{RBF}$ and ${\hat{L}}_{STATIC}$ are the maximum likelihoods for the Gaussian process model and a static model, respectively (Yang et al., 2020). A value of p of 0.05 was deemed significant, but just 25/3515 peaks (0.71%) fulfilled this stringent criterion. A more inclusive threshold of LR < −0.25 was applied to ATAC-seq peaks prior to clustering, representing 7.8% of the total (274/3515). These ATAC-seq peaks were clustered using a Gaussian process mixture model (Hensman et al., 2013). This analysis was done by adapting a code previously produced (Yang et al., 2020) and applying it to our data. Parts of the used code are written in R (R Core Team, 2021), using the ‘gptk’ package (Kalaitzis et al., 2014), and others are written in Python, using the GPy (Sheffield Machine Learning Software, 2012) and GPclust (Hensman et al., 2014) libraries, besides the ones included in the Anaconda data science toolkit (Anaconda Inc., 2020). The original code can be found at the following site: https://github.com/ManchesterBioinference/IntegratingATAC-RNA-HiC/tree/master/ATACseq

### Statistical analysis of peak composition

We computed the percentages of each kind of transcriptional units mapped to our ATAC-seq peaks (mRNA, tRNA, or other), as well as their location within the transcriptional unit (TSS, TES, exon, or intergenic region), and for the fraction of peaks identified as dynamic. We compared the percentages of each class in the dynamic peaks vs. the percentage on rest of the peaks by computing a Z score for two population proportions by the formula $Z=\frac{\left({\bar{p}}_{1}-{\bar{p}}_{2}\right)-0}{\sqrt{\bar{p}\left(1-\bar{p}\right)\left(\frac{1}{n_{1}}+\frac{1}{n_{2}}\right)}}$ , where ${\bar{p}}_{1}$ and ${\bar{p}}_{2}$ are the respective proportions of each population for a particular class, $\bar{p}$ is the combined proportion of that class, and $n_{1}$ and $n_{2}$ are the total number of peaks in each population. The associated two-sided value of *p* was calculated with the *Z* distribution and the change was considered significant if the value of *p* ≤ 0.05.

### Over-representation analysis of dynamic peaks

We explored if any gene set was overrepresented among the peaks clustering in each of the dynamic categories we have identified, using as input the SGD gene IDs associated with the peaks within each category using WebGestalt (Liao et al., 2019). We have selected *Saccharomyces cerevisiae* as the organism of interest and ORA as the analysis method. We explored the three Gene Ontology functional domains (Biological Process, Molecular Function, and Cellular Component) as well as the KEGG pathways database, keeping the values for the advanced parameters at their default levels. Those gene sets with a False Discovery Rate (FDR) smaller or equal to 0.1 were considered significant, which in our case was none.

### Gene set enrichment analysis of ATAC-seq peaks corresponding to differential compaction on consecutive time points

We performed comparisons of the ATAC-seq peaks observed in time-consecutive samples using DESeq2 as described above. For those comparisons with a large number of differentially changed peak values, we have used the SGD gene ID associated with each peak and the log_2_-fold change across samples for those peaks displaying significant changes (*p*_adj_ < 0.05) as input for performing GSEA using WebGestalt (Liao et al., 2019). For genes that were associated with multiple peaks, each peak was separately considered and compared across time points and accounted in the GSEA. We have selected *Saccharomyces cerevisiae* as the organism of interest and GSEA as the analysis method. We explored the three Gene Ontology functional domains (Biological Process, Molecular Function, and Cellular Component) as well as the KEGG pathways database, keeping the advanced parameters at their default levels; we selected to obtain the top5 categories positively or negatively enriched in each of the domains/database. Those gene sets with a False Discovery Rate (FDR) smaller or equal to 0.1 were considered significant.

## Results

### Laboratory evolution of the yeast genome under selection pressure

As part of our recent study, we performed a 28-day-long laboratory evolution experiment by subjecting wild-type yeast cells expressing P_GAL1_-YFP to repeated environmental selection in the form of daily sorting. During the first 7 days, the cells were sorted daily based on the lowest 5% of the YFP expression, and were regrown in the same environment until the next day’s sorting activity. We saw varying degrees of reductions in reporter expression at the end of the 7-day selection period (Luo et al., 2020). These expression reductions persisted for most of the biological replicates during an additional 21-day-long selection-free growth period, showing the stable nature of the expression changes as a result of the selection pressure.

Cloning a second reporter, P*_TEF1_*-mCherry or P*_GAL1_*-mCherry, in the same strain carrying the P_GAL1_-YFP cassette and performing YFP sorting experiments on both strains led to no change in the expression level of mCherry in the P*_TEF1_*-mCherry strain, but significant reduction in the mCherry expression level in the P*_GAL1_*-mCherry strain. Therefore, the reduced expression originally observed was found to be due to factors in the GAL network rather than global factors that could be expected to also affect the expression from P*_TEF1_*-mCherry. We further found that the local chromatin environment of the reporter cassette had a significant effect on the observed phenotype (Luo et al., 2020).

**Figure 2:**
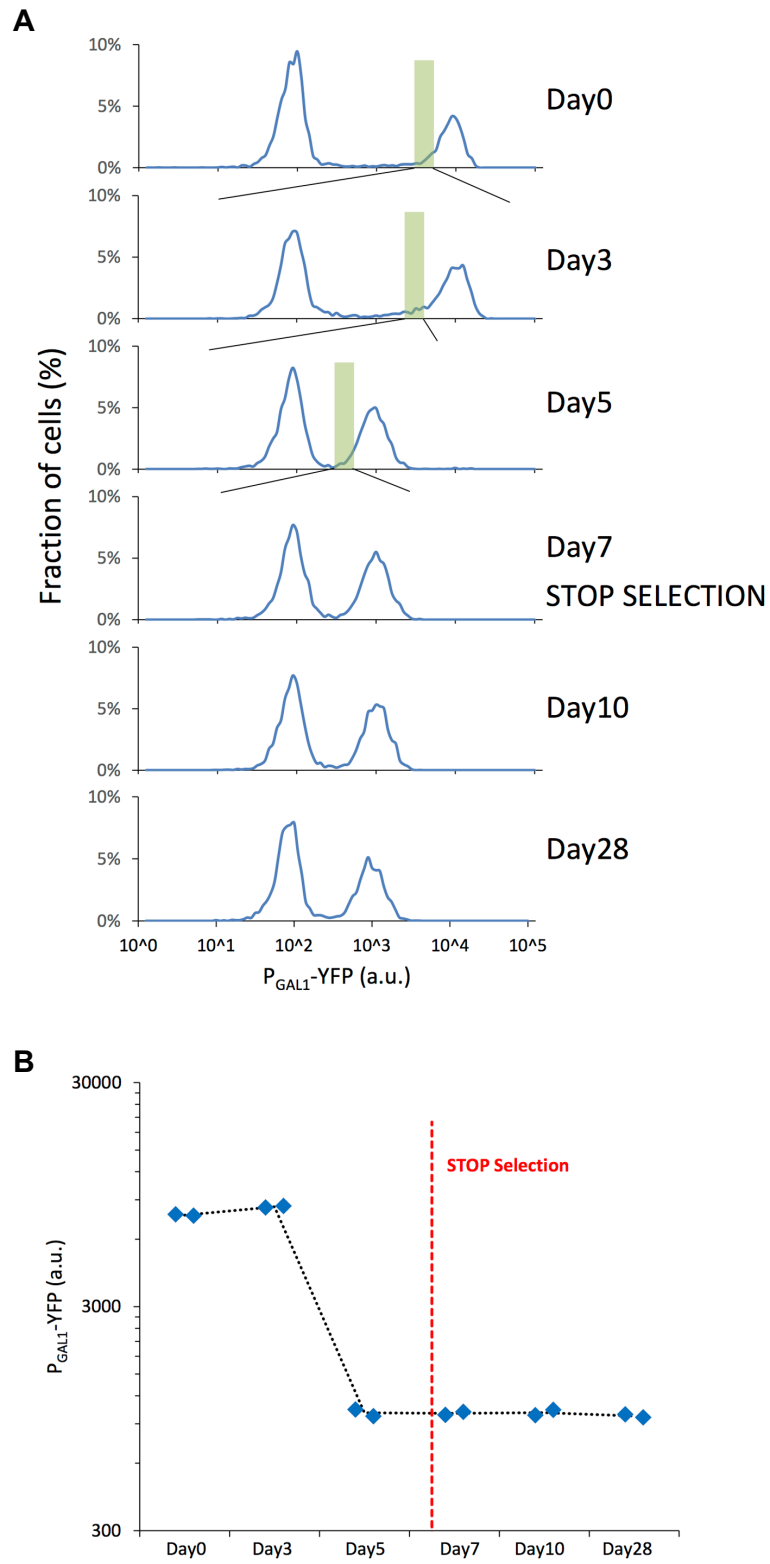
Microevolution experiment with selection of the cells with low YFP expression. **(A)** P_GAL1_-driven YFP expression distributions of the revived yeast cells grown in 0.1% mannose & 0.2% galactose synthetic complete media, indicating the fraction of cells expressing different levels of YFP with cell fractions indicating normalization to the total number of cells plotted in each panel. The expression distributions correspond to the different days of the original evolution experiment. The green rectangle represents the selected/sorted population at each time point, corresponding to the lowest 5% of the YFP-ON cells. The peak left to the gray dashed line corresponds to OFF expression state of the bistable GAL network. **(B)** Mean P_GAL1_-YFP expression of the ON cells was measured on selected days of the microevolution experiment. Blue diamonds represent the values of each replicate. The red dashed line represents the moment when selection pressure was lifted.

To understand how genome-wide chromatin compaction states changed during the course of evolution, we applied the ATAC-seq assay on samples revived from different days of our evolution experiment, including days 0, 3, 7, 10, and 28. The revived expression distributions also displayed the previously-observed expression reduction between days 0 and 7, and stability between days 7 and 28 (Figures 2A,B).

### Global chromatin compaction analysis of the evolving yeast genome under selection pressure

**Figure 3:**
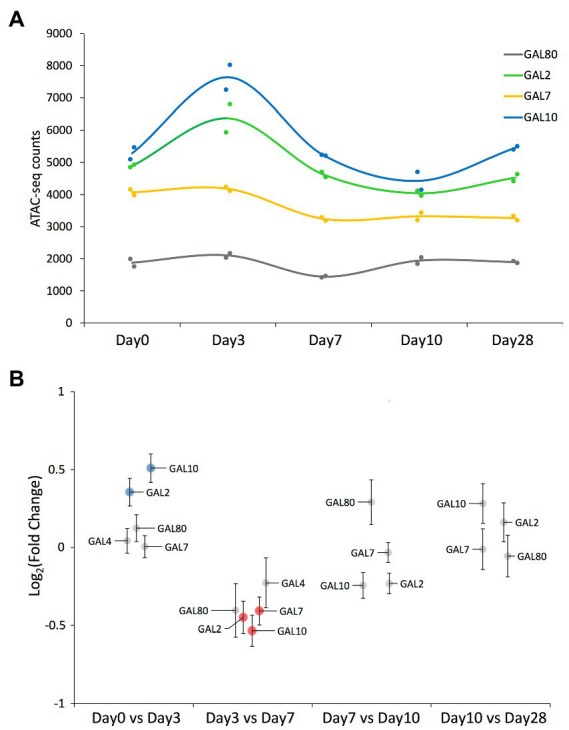
ATAC-seq results on peaks mapped to the GAL network genes. **(A)** ATAC-seq peak value of those peaks mapping to the GAL network genes that have been detected across all time points. Solid circles represent the values corresponding to each replicate, while the smooth line represents the average value at each time point. Each gene’s common name is indicated in the figure panel. **(B)** Swarm plot showing the log_2_-fold change on each of the ATAC-seq peaks mapping to the GAL network genes, comparing samples of consecutive time points. Each peak’s associated gene is indicated by a label (note that GAL4’s associated peak was not detected in the last two time points). Significantly differential peaks (*p*_adj_ < 0.05) are plotted either in blue (if increased in the latter time point) or in red (if increased in the former time point).

We detected ATAC-seq peaks mapping to all GAL network genes across most of the time points (Figures 3A,B; Supplementary Files 1, 2). Chromatin around the GAL2 and GAL10 genes became significantly less compact between days 0 and 3, with the reverse trend observed for GAL2, GAL7, and GAL10 between days 3 and 7. Note that the GAL10 peak was assigned to the promoter region, so it could be equally assigned to GAL1 as both genes share a bidirectional promoter (Lohr, 1997; Elison et al., 2018).

**Figure 4:**
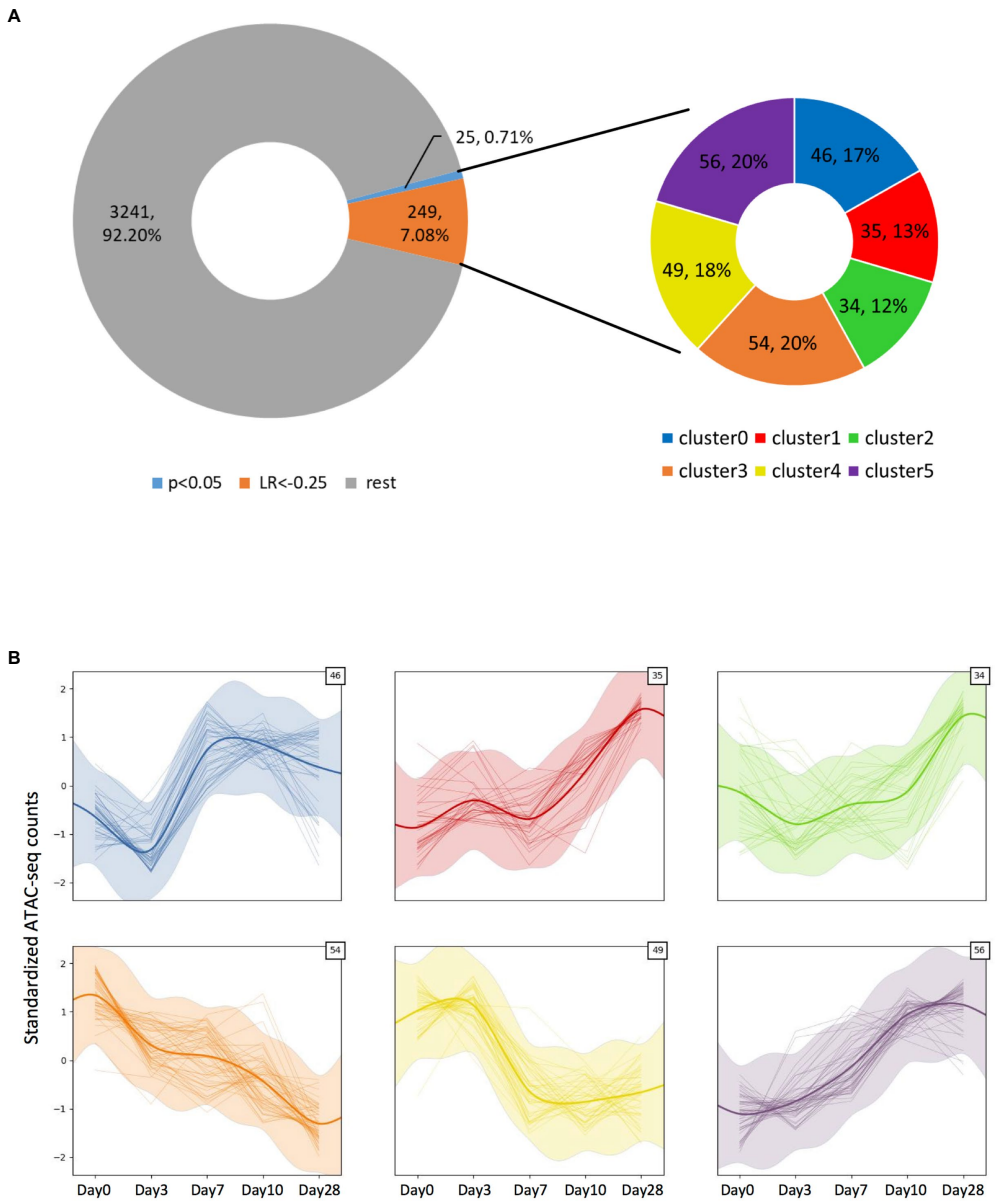
Identification of the ATAC-seq peaks displaying temporal dynamics. **(A)** Doughnut chart representing all significant peaks observed from the ATAC-seq assay. Among them, a small fraction displays significant (value of *p* > 0.05) temporal dynamics as defined by a Gaussian process mixture model, but by applying a log-likelihood ratio-based criteria with LR < −0.25, ~7.8% of the peaks could be considered to have dynamic behavior. The dynamic peaks could be clustered into six categories, depending on their specific dynamic behavior. **(B)** Plots showing each category’s dynamic behavior defined by the Gaussian process mixture model mean and SD (represented by the thick smoothed line and shaded ribbon), as well as each clustered peak count value across time (thin lines). The number on the top-right corner indicates the number of peaks included in each cluster.

Altogether, we detected 3,515 significant ATAC-seq peaks (Figure 4A, Supplementary File 1), and most of them localized at the promoter/TSS regions (Supplementary Figures S1, S2, S3A). To understand if there were any temporal peak dynamics, we applied a Gaussian process mixture model. By applying a very stringent χ^2^ test on the log-likelihood ratio (LR) after fitting the dynamic and static models on the data, just 25 peaks (0.71%) could be considered to have a dynamic trend (value of *p* <0.05). We then used a more permissive threshold of LR < −0.25 to consider a particular fit to be dynamic, leading to the identification of a total of 274 peaks (7.8%). Among those peaks with temporal dynamics, their location within the transcriptional unit was further analyzed and we found them to be overrepresented in the promoters (value of *p* = 3.54E-04, Supplementary Figure S3A). Intriguingly, tRNA transcriptional units were overrepresented in the fraction of dynamic peaks compared to the whole detected peak population (value of *p* = 9.74E-03, Supplementary Figure S3B). Those dynamic peaks could further be clustered in six categories based on their specific dynamic behavior (Figure 4B; Supplementary File 1). We identified the genes associated with each category and wanted to see if any gene set was particularly enriched on those categories by performing Over-Representation Analysis (ORA) on each of the categories. While we explored the biological process, molecular function, and cellular component domains of the Gene Ontology as well as the KEGG pathways, we did not find any gene set significantly overrepresented in any of the categories. Also, no gene set was enriched when we inputted into ORA the genes associated with the 274 dynamic peaks we identified (data not shown).

**Figure 5:**
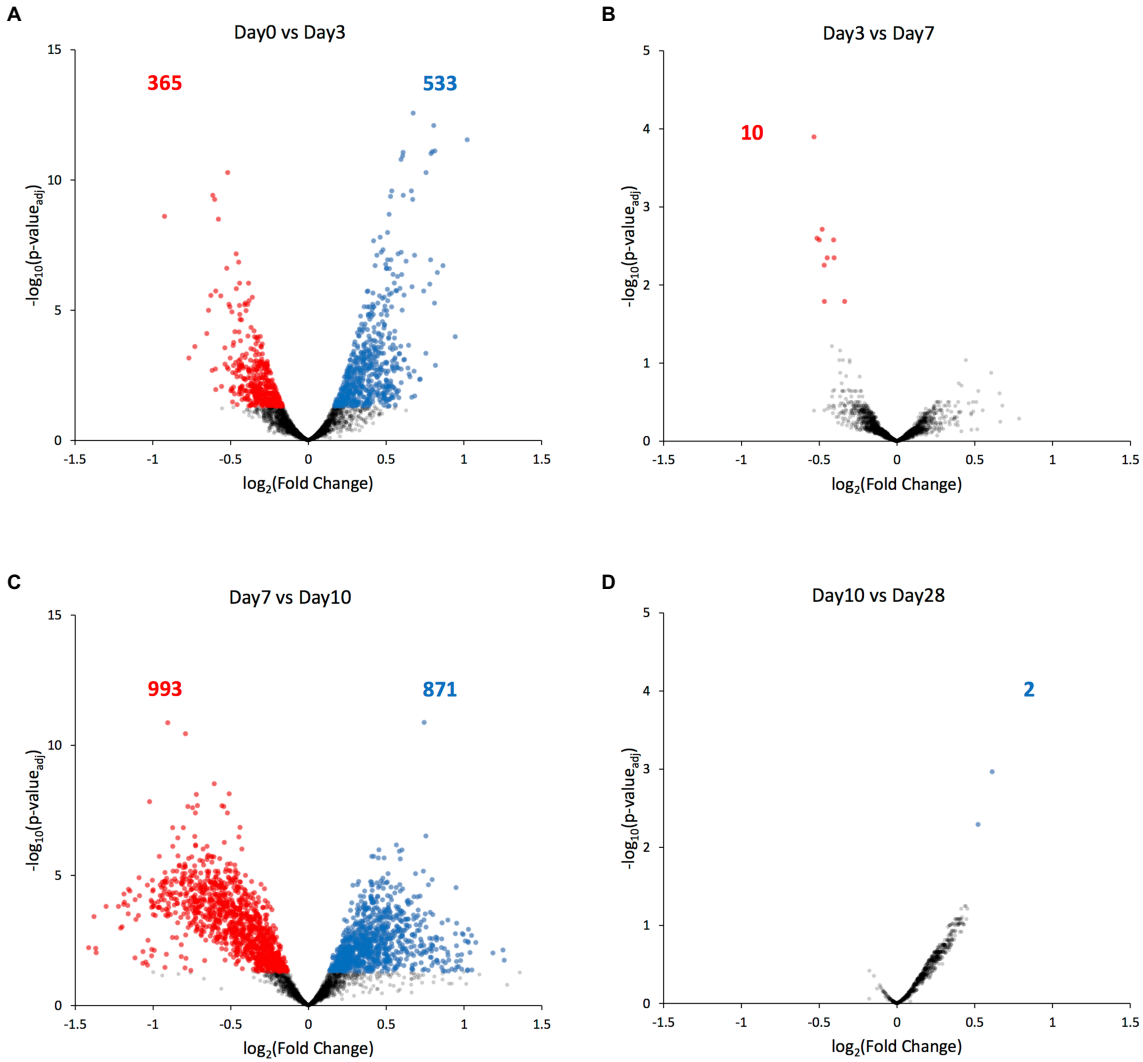
Genes with differentially compacted chromatin between consecutive time points. **(A–D)** Volcano plots comparing each ATAC-seq peak’s value at consecutive time points as a log_2_ fold-change. Significantly differential peaks (*p*_adj_ < 0.05) are plotted either in blue (if increased in the latter time point) or in red (if increased in the former time point). The blue and red numbers indicate the number of genes with significantly altered chromatin compaction between each time point.

Next, we compared each ATAC-seq peak’s value between consecutive days, and identified the significantly differential peaks (*p*_adj_ < 0.05). Most of the significant chromatin compaction changes occurred between days 0 and 3 when the selection pressure was first applied (898 differentially compacted peaks, Figure 5A), and then between days 7 and 10 when the selection pressure was lifted (1864 differentially compacted peaks, Figure 5C). Among these significant compaction differences, the fraction of peaks experiencing increases in chromatin compaction was roughly the same as the fraction of peaks experiencing decreases in chromatin compaction (Figure 5; Supplementary File 2), indicating that the chromatin did not change its compaction state uniformly across the genome. Only ten genes were found to have altered chromatin compaction between days 3 and 7 (while the selection was present), with three of them belonging to the GAL network (GAL2, GAL7, and GAL10; Figures 3B, 5B). Regarding the remaining seven transcriptional units identified, one codes for alanine tRNA, five of them code for uncharacterized or dubious ORFs (YFL063W, YHR217C, YLL066W-B, YLL065W, YLR081W, and YML122C), and the last one for the plasma membrane permease GIT1 which mediates uptake of glycerophosphoinositol and glycerophosphocholine. Between day10 and day28, during which no selection pressure was applied, just 2 transcriptional units were found to have differential accessibility, the hexose transporter HXT7 and a dubious ORF unlikely to code for a protein (YKR040C). Nonetheless, the fold-change in chromatin accessibility on those transcriptional units was quite low (~0.5 in log_2_ scale), indicating that there is no appreciable change on chromatin compaction between the two time points.

### Gene set enrichment analysis on the differentially compacted chromatin loci

**Figure 6:**
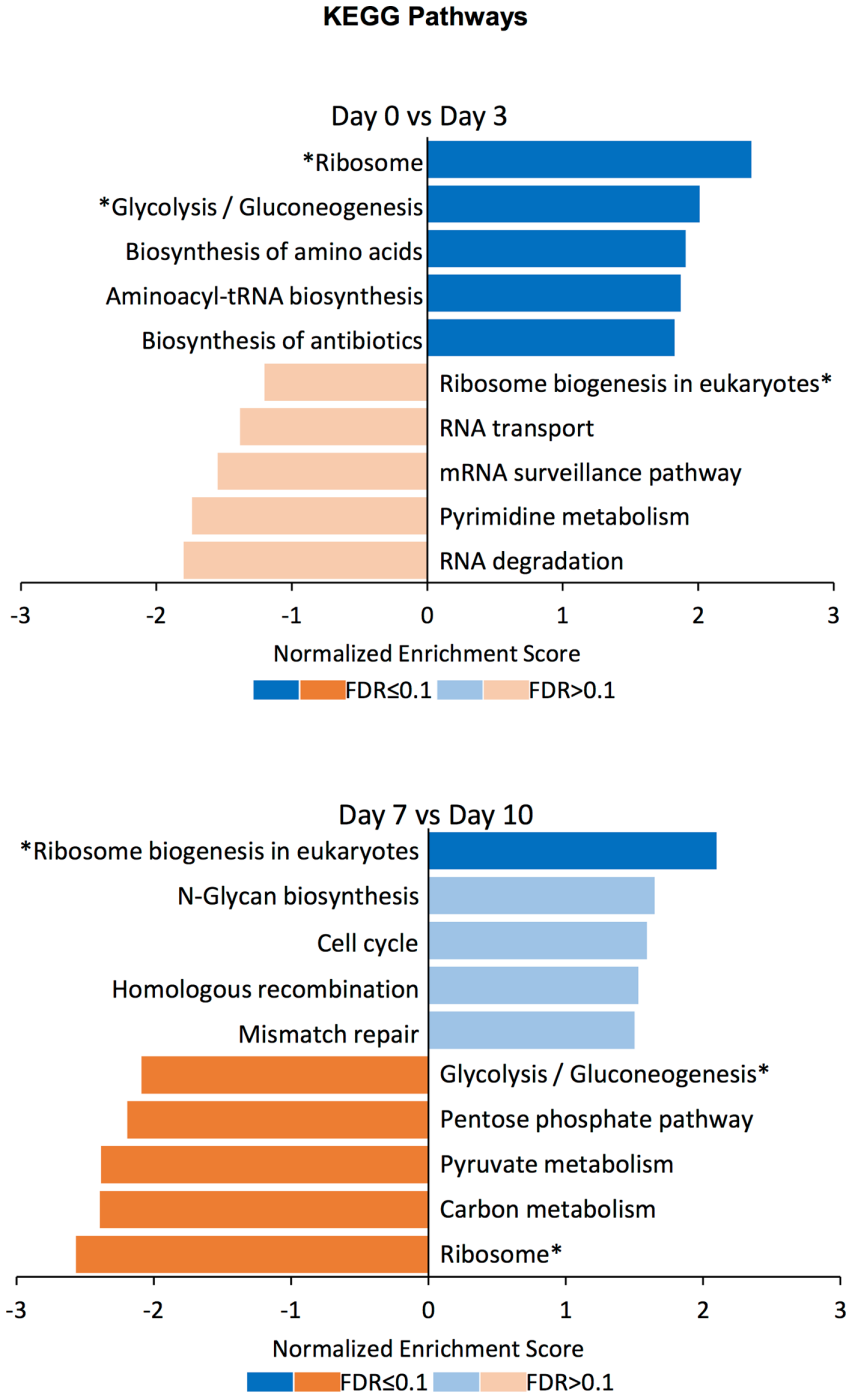
GSEA on differentially compacted loci on the KEGG Pathways domain. Gene Set Enrichment Analysis (GSEA) on the differentially available chromatin loci comparing Day0 vs. Day3 and Day7 vs. Day10 (the two comparisons displaying most of the significant changes genome-wide). The bar plots show the normalized enrichment score for the top5 gene sets with increased (blue) and decreased (orange) chromatin availability in the latter time point. Dark colors indicate an FDR ≤ 0.1 for the indicated gene set, while pale colors indicate an FDR > 0.1. Gene sets that have been identified as increased in the Day0 vs. Day3 comparison and decreased in the Day7 vs. Day10 comparison (or vice versa) are indicated with an asterisk (*).

To find out the gene sets associated with the differentially compacted chromatin loci between the days displaying most of the significant compaction changes, we performed Gene Set Enrichment Analysis (GSEA) between datasets corresponding to days 0 vs. 3, and days 7 vs. 10. We explored gene sets corresponding to the biological process, molecular function, and cellular component domains of the Gene Ontology as well as the KEGG pathways. Normalized enrichment scores for the top5 gene sets with increased or decreased chromatin compaction between these datasets were calculated (Figure 6; Supplementary Figures S4–S6; Supplementary Table S2). Corresponding to the significantly enriched gene sets, there were categories such as ‘mitochondrial gene expression’, ‘methylation’, ‘RNA modification’, and ‘generation of precursor metabolites and energy’, meaning that the changes in chromatin compaction affected a wide range of processes and pathways within the cell. Interestingly, some of the gene sets that have been identified as increased between the Day0 vs. Day3 comparison were decreased in enrichment between the Day7 vs. Day10 comparison (or vice versa); ‘methylation’ in the GO term Biological Process domain and ‘glycolysis/gluconeogenesis’ among the KEGG pathways categories can be given as two examples. Such ‘reversible’ gene sets were present in each of the GO term domains and KEGG pathways, but those categories were not always significantly enriched at the FDR level. Nonetheless, the fact that the same enrichment categories are found on the opposite ends of the spectrum (from high to low levels of chromatin accessibility) both at the beginning and end of the selection process is very intriguing, even if not all categories were scored with a significant FDR. We note that the reversibility is not a global behavior applicable to all genes. It is only achievable by certain genes or gene sets, with other enriched gene sets not displaying reversible behavior across the different periods of the evolution experiment. We expect future research to mechanistically uncover the beneficial impact of displaying reversibility by certain gene groups.

## Discussion

In a previous study, based on a daily selection/enrichment of a particular gene expression state, we performed a microevolution experiment and observed sustained reductions on gene expression that turned out to be contributed by epigenetic changes on key GAL network components (Luo et al., 2020). Using the ATAC-seq assay to directly explore genome-wide chromatin compaction states, here we report chromatin compaction state changes associated with the previously-observed expression reductions. Less accessible chromatin accompanied the reduced GAL expression profiles of the evolved yeast populations. Despite the expected nature of this result, which also validate the application of the ATAC-seq assay, many loci with no relation with the GAL network surprisingly displayed changes in their chromatin compaction states.

Throughout the microevolution, a small fraction of the chromatin displayed dynamic behavior regarding the changes on its compaction state. This was experienced by genes that were not functionally related to each other, nor physically close at the DNA strand level, suggesting that this behavior was randomly formed. From a gene’s “selfish” perspective under selection pressure (Dawkins, 1976; Ågren, 2016), this may constitute a bet-hedging strategy adopted by some genes in order to be better adapt to environmental changes, either to their own benefit or to the benefit of the whole organism.

An interesting outcome of our study is that the genome-wide chromatin compaction changes do not occur synchronously with the changes on the phenotype under artificial selection, but apparently in response to the selection pressure itself. With Day7 being the day on which the selection pressure was lifted, among the time points for which we have collected ATAC-seq data (Day0, Day3, Day7, Day10, and Day28), the vast majority of significant changes on chromatin compaction were detected between Day0 and Day3, and between Day7 and Day10, precisely after the selection pressure was applied and lifted. On the other hand, between Day3 and Day7 (when selection pressure was still being applied) and between Day10 and Day28 (when selection pressure was no longer applied), the number of peaks displaying significant changes was negligible (Figure 5). The lack of changes in chromatin compaction state between the two final time points (Day10 and Day28) also serves as an internal control, as in these two selection-free regimes, one would not expect to see changes in chromatin compaction; this observation supports the validity of the changes we have observed across the earlier time points.

**Figure 7:**
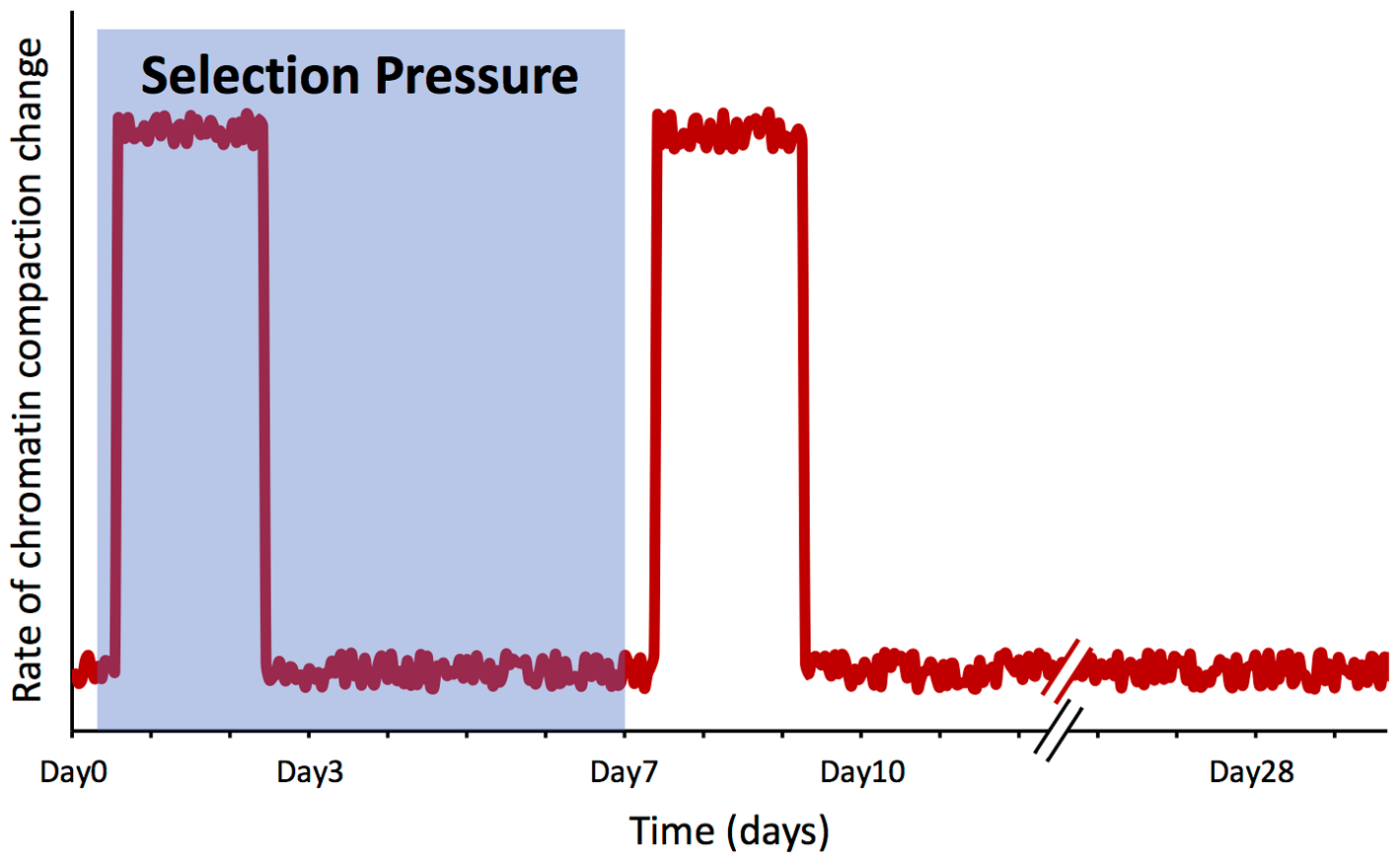
Changes in chromatin compaction spikes in response to selection pressure shifts. Qualitative plot summarizing our findings, representing the rate of change of the chromatin compaction across the genome throughout the microevolution experiment. The shaded area represents the timeframe while selection pressure was applied on the cell population.

These findings point toward a model where the cell population is ‘aware’ of the selection process, triggering a ‘wave’ of chromatin remodeling changes, with first one occurring upon the application of the selection pressure and another one when the selection is over (Figure 7). However, the question of how a non-communicating single cell in a well-mixed population might become aware of this selection is not trivial. Using the same growth media and conditions, the only difference between a cell experiencing the under-selection and selection-free periods was that in the former, a narrow gate (based on P_GAL1_-YFP expression) was imposed to sort cells, while in the selection-free period, this gate was much wider to sort the whole population. It is possible that cell-to-cell variability in chromatin compaction is actually greater at the single-cell level than what we measured here at the population level (validation of this is challenging as single-cell ATAC-seq (Cusanovich et al., 2015) does not exist for yeast yet). Assuming that there is a high degree of noise associated with the chromatin compaction state, if specific YFP expression ranges are associated with certain chromatin compaction states, then sorting cells at a narrow YFP expression range would remove the rest of the population variability from the sample, resulting in a distinct chromatin compaction state during the selection period for the subpopulation being repeatedly selected. Once the sorting-gate gets wider to cover the whole population during the selection-free regime after the 7^th^ day, all the original variability of the population would be captured again; despite the lower P_GAL1_-YFP expression, this would lead to another chromatin compaction profile similar to the one of the initial population before the selection pressure or sorting was applied. This ‘wave’ model matches what we observed experimentally using population-level ATAC-seq.

The two waves of chromatin changes appear to be opposite to each other, at least partially; among the gene set categories enriched when chromatin compaction was increased upon enforcing selection pressure, some became decreased in compaction once the selection was lifted. This would suggest that those categories changed their chromatin compaction during the time when the selection was present, returning to their basal state afterward. By displaying a degree of reversibility on the gene set categories experiencing chromatin compaction changes, the evolving system also showed a type of memory; actually, memory was already present in the system due to the stably-maintained low GAL expression levels throughout the after-selection period, as previously reported (Luo et al., 2020).

The gene set categories representing the genes whose chromatin compaction state significantly changed are very diverse and apparently not related to each other, suggesting that those changes arise randomly, possibly as a result of the cells trying to generate variability to better adapt to the selection pressure. In this case, while all those genome-wide chromatin changes had a neutral effect on the phenotype under selection, a population could try to maximize survival in a competitive environment by utilizing all available tools to produce variability; epigenetic mechanisms and phenotypic changes rooted by epigenetics play an important role in that process, as well as genetic mutations (Day and Bonduriansky, 2011).

Our work represents an example to how environmental selection of a particular trait can be accompanied by a plethora of unexpected changes, in this case at the chromatin compaction level. Future studies focusing on the real-time evolution of microbial populations would show the generality of our findings and provide insights into the physiological relevance of the genome-wide chromatin compaction changes experienced by eukaryotic genomes.

## Data availability statement

The data presented in the study are deposited in the NCBI GEO repository, accession number GSE205227, https://www.ncbi.nlm.nih.gov/geo/query/acc.cgi?acc=GSE205227.

## Author contributions

DM and MA designed the experiments and analyses, interpreted the data and results, and designed and prepared the manuscript. DM performed experiments, and collected and analyzed the data. MA conceived and supervised the project. All authors contributed to the article and approved the submitted version.

## Funding

MA acknowledges funding from the National Institutes of Health (5R01GM127870).

## Conflict of interest

The authors declare that the research was conducted in the absence of any commercial or financial relationships that could be construed as a potential conflict of interest.

## Publisher’s note

All claims expressed in this article are solely those of the authors and do not necessarily represent those of their affiliated organizations, or those of the publisher, the editors and the reviewers. Any product that may be evaluated in this article, or claim that may be made by its manufacturer, is not guaranteed or endorsed by the publisher.

## Supplementary material

TABLE 2 Supplementary Table S2.

PRESENTATION 1 Supplementary Figures S1-S6, Supplementary Table legends and Supplementary File legends.

DATA SHEET 1 Supplementary File 1.

DATA SHEET 2 Supplementary File 2.

## References

[ref1] Acar M. Becskei A. van Oudenaarden A. (2005). Enhancement of cellular memory by reducing stochastic transitions. Nature 435, 228–232. doi: 10.1038/nature03524, PMID: 15889097

[ref2] Acar M. Mettetal J. T. van Oudenaarden A. (2008). Stochastic switching as a survival strategy in fluctuating environments. Nat. Genet. 40, 471–475. doi: 10.1038/ng.110, PMID: 18362885

[ref3] Acar M. Pando B. F. Arnold F. H. Elowitz M. B. van Oudenaarden A. (2010). A general mechanism for network-dosage compensation in gene circuits. Science 329, 1656–1660. doi: 10.1126/science.1190544, PMID: 20929850 PMC3138731

[ref4] Ågren J. A. (2016). Selfish genetic elements and the gene’s-eye view of evolution. Curr. Zool. 62, 659–665. doi: 10.1093/cz/zow102, PMID: 29491953 PMC5804262

[ref5] Anaconda Inc. (2020). Anaconda Software Distribution. Available at: https://docs.anaconda.com/ (Accessed April 10, 2022).

[ref6] Bintu L. Yong J. Antebi Y. E. McCue K. Kazuki Y. Uno N.. (2016). Dynamics of epigenetic regulation at the single-cell level. Science 351, 720–724. doi: 10.1126/science.aab2956, PMID: 26912859 PMC5108652

[ref7] Bódi Z. Farkas Z. Nevozhay D. Kalapis D. Lázár V. Csörgő B.. (2017). Phenotypic heterogeneity promotes adaptive evolution. PLoS Biol. 15, e2000644–e2000626. doi: 10.1371/journal.pbio.2000644, PMID: 28486496 PMC5423553

[ref8] Bonduriansky R. Day T. (2009). Nongenetic inheritance and its evolutionary implications. Annu. Rev. Ecol. Evol. Syst. 40, 103–125. doi: 10.1146/annurev.ecolsys.39.110707.173441

[ref9] Buenrostro J. D. Giresi P. G. Zaba L. C. Chang H. Y. Greenleaf W. J. (2013). Transposition of native chromatin for fast and sensitive epigenomic profiling of open chromatin, DNA-binding proteins and nucleosome position. Nat. Methods 10, 1213–1218. doi: 10.1038/nmeth.2688, PMID: 24097267 PMC3959825

[ref10] Burggren W. (2014). Epigenetics as a source of variation in comparative animal physiology – or – Lamarck is lookin’ pretty good these days. J. Exp. Biol. 217, 682–689. doi: 10.1242/jeb.086132, PMID: 24574384

[ref11] Chatterjee M. Acar M. (2018). Heritable stress response dynamics revealed by single-cell genealogy. Sci. Adv. 4:e1701775. doi: 10.1126/sciadv.1701775, PMID: 29675464 PMC5906080

[ref12] Cusanovich D. A. Daza R. Adey A. Pliner H. A. Christiansen L. Gunderson K. L.. (2015). Multiplex single cell profiling of chromatin accessibility by combinatorial cellular indexing. Science 348, 910–914. doi: 10.1126/science.aab1601, PMID: 25953818 PMC4836442

[ref13] D’Urso A. Brickner J. H. (2014). Mechanisms of epigenetic memory. Trends Genet. 30, 230–236. doi: 10.1016/j.tig.2014.04.004, PMID: 24780085 PMC4072033

[ref14] Dawkins R. (1976). The Selfish Gene. Oxford: Oxford University Press.

[ref15] Day T. Bonduriansky R. (2011). A unified approach to the evolutionary consequences of genetic and nongenetic inheritance. Am. Nat. 178, E18–E36. doi: 10.1086/660911, PMID: 21750377

[ref16] Elison G. L. Xue Y. Song R. Acar M. (2018). Insights into bidirectional gene expression control using the canonical GAL1/GAL10 promoter. Cell Rep. 25, 737–748.e4. doi: 10.1016/j.celrep.2018.09.050, PMID: 30332652 PMC6263159

[ref17] Feng J. Liu T. Qin B. Zhang Y. Liu X. S. (2012). Identifying ChIP-seq enrichment using MACS. Nat. Protoc. 7, 1728–1740. doi: 10.1038/nprot.2012.101, PMID: 22936215 PMC3868217

[ref18] Friman E. T. Deluz C. Meireles-Filho A. C. Govindan S. Gardeux V. Deplancke B.. (2019). Dynamic regulation of chromatin accessibility by pluripotency transcription factors across the cell cycle. elife 8:e50087. doi: 10.7554/eLife.50087, PMID: 31794382 PMC6890464

[ref19] Halfmann R. Jarosz D. F. Jones S. K. Chang A. Lancaster A. K. Lindquist S. (2012). Prions are a common mechanism for phenotypic inheritance in wild yeasts. Nature 482, 363–368. doi: 10.1038/nature10875, PMID: 22337056 PMC3319070

[ref20] Heinz S. Benner C. Spann N. Bertolino E. Lin Y. C. Laslo P.. (2010). Simple combinations of lineage-determining transcription factors prime cis-regulatory elements required for macrophage and B cell identities. Mol. Cell 38, 576–589. doi: 10.1016/j.molcel.2010.05.004, PMID: 20513432 PMC2898526

[ref21] Hendrickson D. G. Soifer I. Wranik B. J. Botstein D. Scott McIsaac R. (2018a). Simultaneous profiling of DNA accessibility and gene expression dynamics with ATAC-Seq and RNA-Seq. Methods Mol. Biol. 819, 317–333. doi: 10.1007/978-1-4939-8618-7_15 30421411

[ref22] Hendrickson D. G. Soifer I. Wranik B. J. Kim G. Robles M. Gibney P. A.. (2018b). A new experimental platform facilitates assessment of the transcriptional and chromatin landscapes of aging yeast. elife 7:74. doi: 10.7554/eLife.39911, PMID: 30334737 PMC6261268

[ref23] Hensman J. Lawrence N. D. Rattray M. (2013). Hierarchical Bayesian modelling of gene expression time series across irregularly sampled replicates and clusters. BMC Bioinformat. 14:252. doi: 10.1186/1471-2105-14-252, PMID: 23962281 PMC3766667

[ref24] Hensman J. Svensson V. Zwiessele M. Lawrence N. D. (2014). GPclust. Available at: https://github.com/SheffieldML/GPclust (Accessed April 10, 2022).

[ref25] Huang S. (2009). Non-genetic heterogeneity of cells in development: more than just noise. Development 136, 3853–3862. doi: 10.1242/dev.035139, PMID: 19906852 PMC2778736

[ref26] Iwasaki M. Paszkowski J. (2014). Epigenetic memory in plants. EMBO J. 33, 1987–1998. doi: 10.15252/embj.201488883, PMID: 25104823 PMC4195768

[ref27] Kakebeen A. D. Chitsazan A. D. Williams M. C. Saunders L. M. Wills A. E. (2020). Chromatin accessibility dynamics and single cell RNA-Seq reveal new regulators of regeneration in neural progenitors. elife 9. doi: 10.7554/eLife.52648, PMID: 32338593 PMC7250574

[ref28] Kalaitzis A. Honkela A. Gao P. Lawrence N. D. (2014). gptk: Gaussian Processes Tool-Kit. Available at: https://cran.r-project.org/package=gptk (Accessed April 10, 2022).

[ref29] Krueger F. James F. Ewels P. Afyounian E. Schuster-Boeckler B. (2018). Trim Galore. doi:10.5281/zenodo.5127899

[ref30] Langmead B. Salzberg S. L. (2012). Fast gapped-read alignment with Bowtie 2. Nat. Methods 9, 357–359. doi: 10.1038/nmeth.1923, PMID: 22388286 PMC3322381

[ref31] Liao Y. Smyth G. K. Shi W. (2014). Feature counts: an efficient general purpose program for assigning sequence reads to genomic features. Bioinformatics 30, 923–930. doi: 10.1093/bioinformatics/btt656, PMID: 24227677

[ref32] Liao Y. Wang J. Jaehnig E. J. Shi Z. Zhang B. (2019). Web gestalt 2019: gene set analysis toolkit with revamped UIs and APIs. Nucleic Acids Res. 47, W199–W205. doi: 10.1093/nar/gkz401, PMID: 31114916 PMC6602449

[ref33] Lohr D. (1997). Nucleosome transactions on the promoters of the yeast GAL and PHO genes. J. Biol. Chem. 272, 26795–26798. doi: 10.1074/jbc.272.43.26795, PMID: 9341105

[ref34] Love M. I. Huber W. Anders S. (2014). Moderated estimation of fold change and dispersion for RNA-seq data with DESeq2. Genome Biol. 15:550. doi: 10.1186/s13059-014-0550-8, PMID: 25516281 PMC4302049

[ref35] Luo X. Song R. Acar M. (2018). Multi-component gene network design as a survival strategy in diverse environments. BMC Syst. Biol. 12:85. doi: 10.1186/s12918-018-0609-3, PMID: 30257679 PMC6158886

[ref36] Luo X. Song R. Moreno D. F. Ryu H. Y. Hochstrasser M. Acar M. (2020). Epigenetic mechanisms contribute to evolutionary adaptation of gene network activity under environmental selection. Cell Rep. 33:108306. doi: 10.1016/j.celrep.2020.108306, PMID: 33113358 PMC7656290

[ref37] Meir Z. Mukamel Z. Chomsky E. Lifshitz A. Tanay A. (2020). Single-cell analysis of clonal maintenance of transcriptional and epigenetic states in cancer cells. Nat. Genet. 52, 709–718. doi: 10.1038/s41588-020-0645-y, PMID: 32601473 PMC7610382

[ref38] Melfi M. D. Lasker K. Zhou X. Shapiro L. (2021). ATAC-seq reveals megabase-scale domains of a bacterial nucleoid. bio Rxiv. doi: 10.1101/2021.01.09.426053

[ref39] Migicovsky Z. Kovalchuk I. (2011). Epigenetic memory in mammals. Front. Genet. 2. doi: 10.3389/fgene.2011.00028, PMID: 22303324 PMC3268583

[ref40] Peng W. Liu P. Xue Y. Acar M. (2015). Evolution of gene network activity by tuning the strength of negative-feedback regulation. Nat. Commun. 6:6226. doi: 10.1038/ncomms7226, PMID: 25670371

[ref41] Quinlan A. R. Hall I. M. (2010). BEDTools: a flexible suite of utilities for comparing genomic features. Bioinformatics 26, 841–842. doi: 10.1093/bioinformatics/btq033, PMID: 20110278 PMC2832824

[ref42] R Core Team (2021). R: A Language and Environment for Statistical Computing. Available at: https://www.r-project.org/ (Accessed January 15, 2022).

[ref43] Ronin I. Katsowich N. Rosenshine I. Balaban N. Q. (2017). A long-term epigenetic memory switch controls bacterial virulence bimodality. elife 6:e19599. doi: 10.7554/eLife.19599, PMID: 28178445 PMC5295817

[ref44] Sheffield Machine Learning Software (2012). GPy: A Gaussian Process Framework in Python. Available at: https://github.com/SheffieldML/GPy (Accessed April 10, 2022).

[ref45] Skinner M. K. (2015). Environmental epigenetics and a unified theory of the molecular aspects of evolution: a neo-Lamarckian concept that facilitates neo-Darwinian evolution. Genome Biol. Evol. 7, 1296–1302. doi: 10.1093/gbe/evv073, PMID: 25917417 PMC4453068

[ref46] Skinner M. K. Guerrero-Bosagna C. Haque M. M. (2015). Environmentally induced epigenetic transgenerational inheritance of sperm epimutations promote genetic mutations. Epigenetics 10, 762–771. doi: 10.1080/15592294.2015.1062207, PMID: 26237076 PMC4622673

[ref47] Stajic D. Perfeito L. Jansen L. E. T. (2019). Epigenetic gene silencing alters the mechanisms and rate of evolutionary adaptation. Nat. Ecol. Evol. 3, 491–498. doi: 10.1038/s41559-018-0781-2, PMID: 30718851

[ref48] Tyedmers J. Madariaga M. L. Lindquist S. (2008). Prion switching in response to environmental stress. PLoS Biol. 6:e294. doi: 10.1371/journal.pbio.0060294, PMID: 19067491 PMC2586387

[ref49] Van Rechem C. Ji F. Chakraborty D. Black J. C. Sadreyev R. I. Whetstine J. R. (2021). Collective regulation of chromatin modifications predicts replication timing during cell cycle. Cell Rep. 37:109799. doi: 10.1016/j.celrep.2021.109799, PMID: 34610305 PMC8530517

[ref50] Xue Y. Acar M. (2018a). Live-cell imaging of chromatin condensation dynamics by CRISPR. iScience 4, 216–235. doi: 10.1016/j.isci.2018.06.001, PMID: 30027155 PMC6048970

[ref51] Xue Y. Acar M. (2018b). Mechanisms for the epigenetic inheritance of stress response in single cells. Curr. Genet. 64, 1221–1228. doi: 10.1007/s00294-018-0849-1, PMID: 29846762 PMC6215725

[ref52] Yang J. McGovern A. Martin P. Duffus K. Ge X. Zarrineh P.. (2020). Analysis of chromatin organization and gene expression in T cells identifies functional genes for rheumatoid arthritis. Nat. Commun. 11:4402. doi: 10.1038/s41467-020-18180-7, PMID: 32879318 PMC7468106

[ref53] Yang J. Penfold C. A. Grant M. R. Rattray M. (2016). Inferring the perturbation time from biological time course data. Bioinformatics 32, 2956–2964. doi: 10.1093/bioinformatics/btw329, PMID: 27288495 PMC5039917

