# Supplementary material for: Phenotypic selection during laboratory evolution of yeast populations leads to a genome-wide sustainable chromatin compaction shift
Source: Front Microbiol. 2022 Oct 13;13:974055. doi: 10.3389/fmicb.2022.974055 (PMC9615041; doi:10.3389/fmicb.2022.974055)
Supplement: PRESENTATION 1 — Supplementary Figures S1-S6, Supplementary Table legends and Supplementary File legends. [file Presentation_1.PDF]

SUPPLEMENTARY FIGURES and LEGENDS

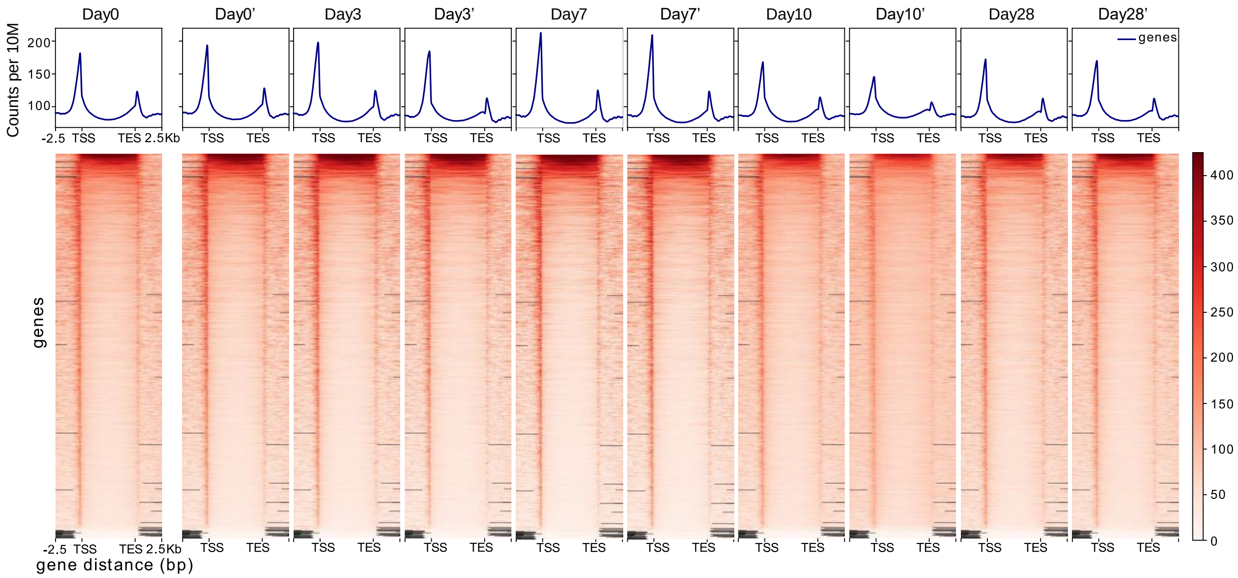

Figure S1. ORF-aligned ATAC-seq reads density. Heat map and general profile of the ATAC-seq reads distribution over the yeast genes (open reading frames) in each of our samples, scaling the ORF length to align both the Transcription Start Site (TSS) and Transcription End Site (TES) in all genes. The color bar represents the density of reads in a given region. The y-axes of the line plots show normalized read density (counts per 10M reads). The x-axes of both the line plot and heatmap indicate each gene's TSS and TES as well as the 2.5kb region before the TSS and after the TES; for space-limitations, the label has just been indicated on the first panel, but the same x-axis label applies to all panels. On top of each line plot, we show the day/replicate information for each ATAC-seq sample (e.g. Day0 and Day0' for the two replicates of Day0). The black lines in the heatmaps indicate sections where there is no read coverage.

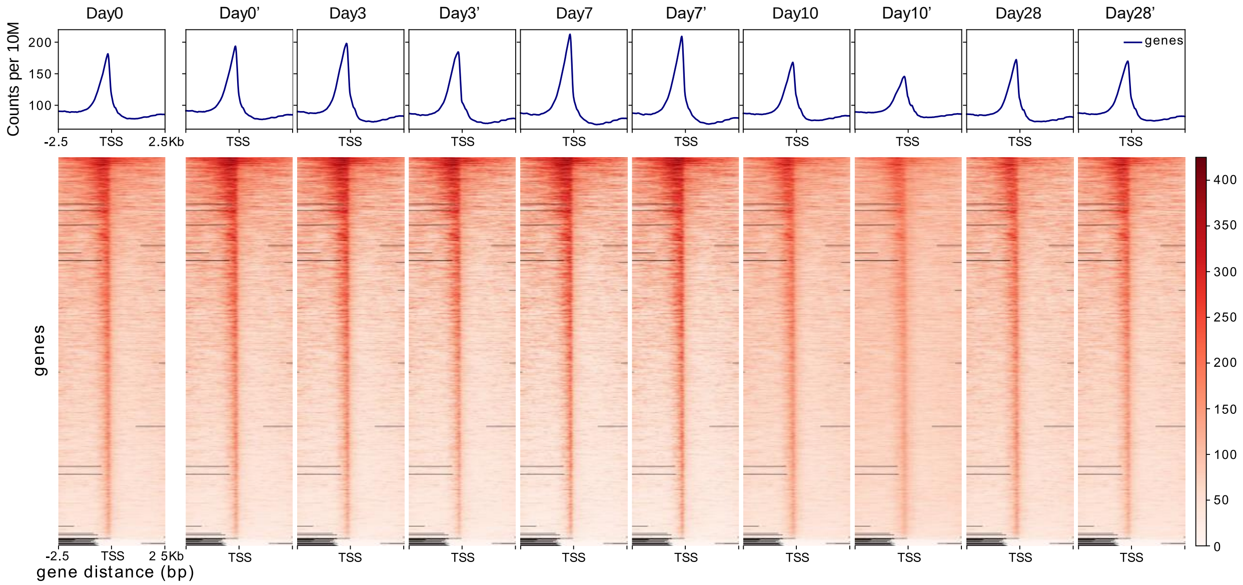

Figure S2. TSS-aligned ATAC-seq reads density. Heat map and general profile of the ATAC-seq reads distribution over the yeast genes in each of our samples, aligning all genes at their Transcription Start Site (TSS). The color bar represents the density of reads in a given region. The y-axes of the line plots show normalized read density (counts per 10M reads). The x-axes of both the line plot and heatmap indicate each gene’s TSS as well as the 2.5kb region before and after the TSS; for space-limitations, the label has just been indicated on the first panel, but the same x-axis label applies to all panels. On top of each line plot, we show the day/replicate information for each ATAC-seq sample (e.g. Day0 and Day0’ for the two replicates of Day0). The black lines in the heatmaps indicate sections where there is no read coverage.

A

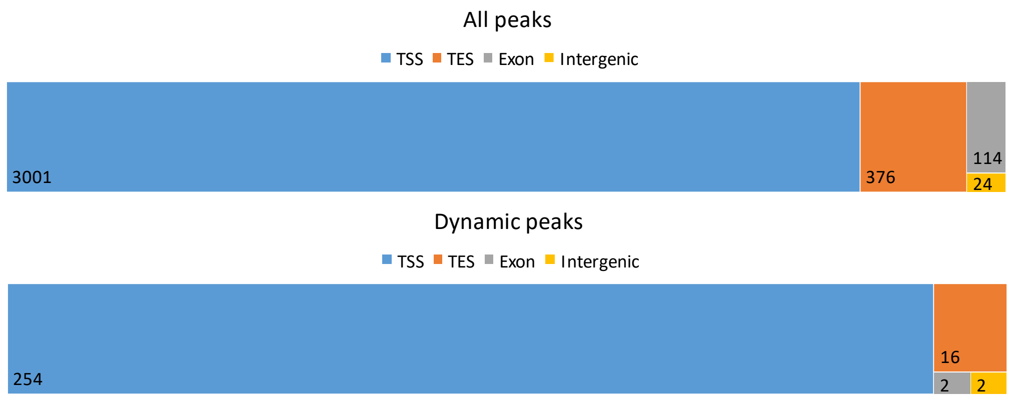

B

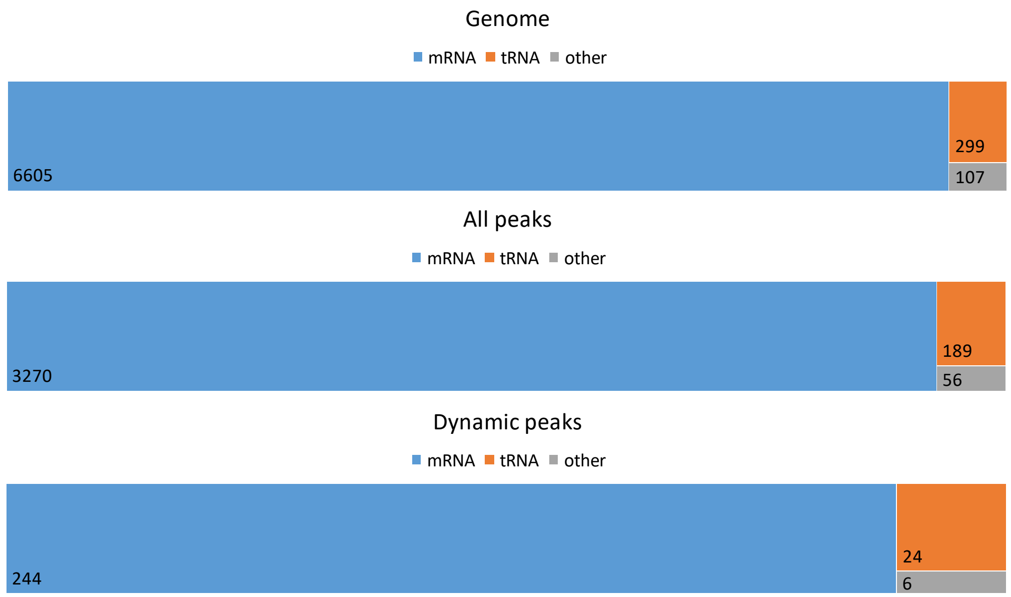

Figure S3. Composition of the genomic loci assigned to the detected ATAC-seq peaks. A. Treemaps representing which ATAC-seq peak locations within transcriptional units (TSS, TES, exon or intragenic regions) are mapped to each of the significant peaks identified by our ATAC-seq analysis, in comparison to the ATAC-seq peaks identified as dynamic (LR < -0.25). The area of each rectangle is proportional to the percentage of each category, and the absolute number of items within each category is indicated. **B.** Treemaps representing which kind of transcriptional units (mRNA, tRNA or other) are mapped to each of the significant peaks identified by our ATAC-seq analysis, in comparison to the composition of the whole genome, as well as the composition of the ATAC-seq peaks identified as dynamic (LR < -0.25). The area of each rectangle is proportional to the percentage of each category, and the absolute number of items within each category is indicated.

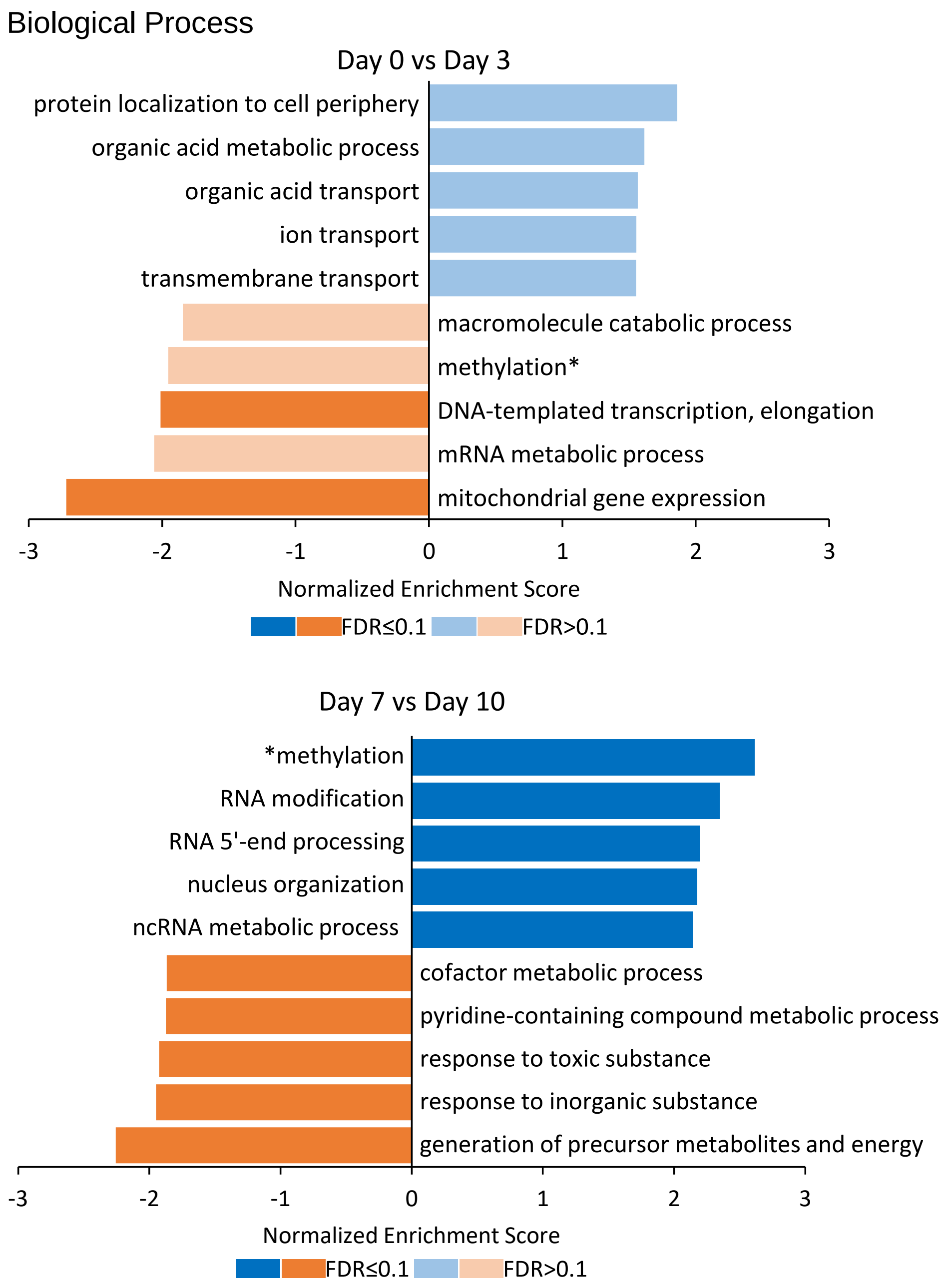

Figure S4. GSEA on differentially compacted loci on the Biological Process GO domain. Gene Set Enrichment Analysis (GSEA) on the differentially available chromatin loci comparing Day0 vs Day3 and Day7 vs Day10 (the two comparisons displaying most of the significant changes genome-wide). The bar plots show the normalized enrichment score for the top5 gene sets with increased (blue) and decreased (orange) chromatin availability in the latter time point. Dark colors indicate an FDR≤0.1 for the indicated gene set, while pale colors indicate an FDR>0.1. Gene sets that have been identified as increased in the Day0 vs Day3 comparison and decreased in the Day7 vs Day10 comparison (or *vice versa*) are indicated with an asterisk (*).

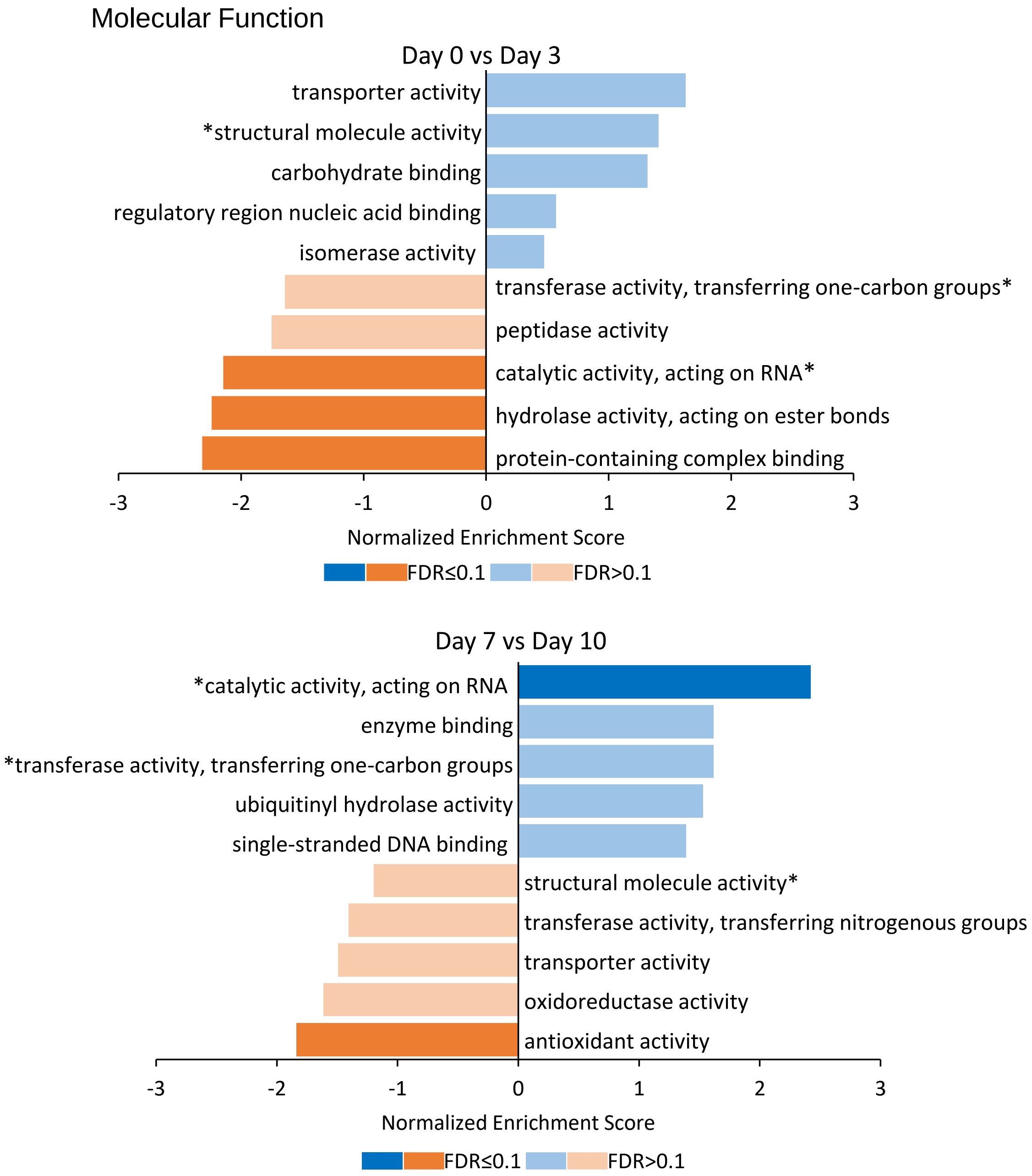

Figure S5. GSEA on differentially compacted loci on the Molecular Function GO domain. Gene Set Enrichment Analysis (GSEA) on the differentially available chromatin loci comparing Day0 vs Day3 and Day7 vs Day10 (the two comparisons displaying most of the significant changes genome-wide). The bar plots show the normalized enrichment score for the top5 gene sets with increased (blue) and decreased (orange) chromatin availability in the latter time point. Dark colors indicate an FDR≤0.1 for the indicated gene set, while pale colors indicate an FDR>0.1. Gene sets that have been identified as increased in the Day0 vs Day3 comparison and decreased in the Day7 vs Day10 comparison (or *vice versa*) are indicated with an asterisk (*).

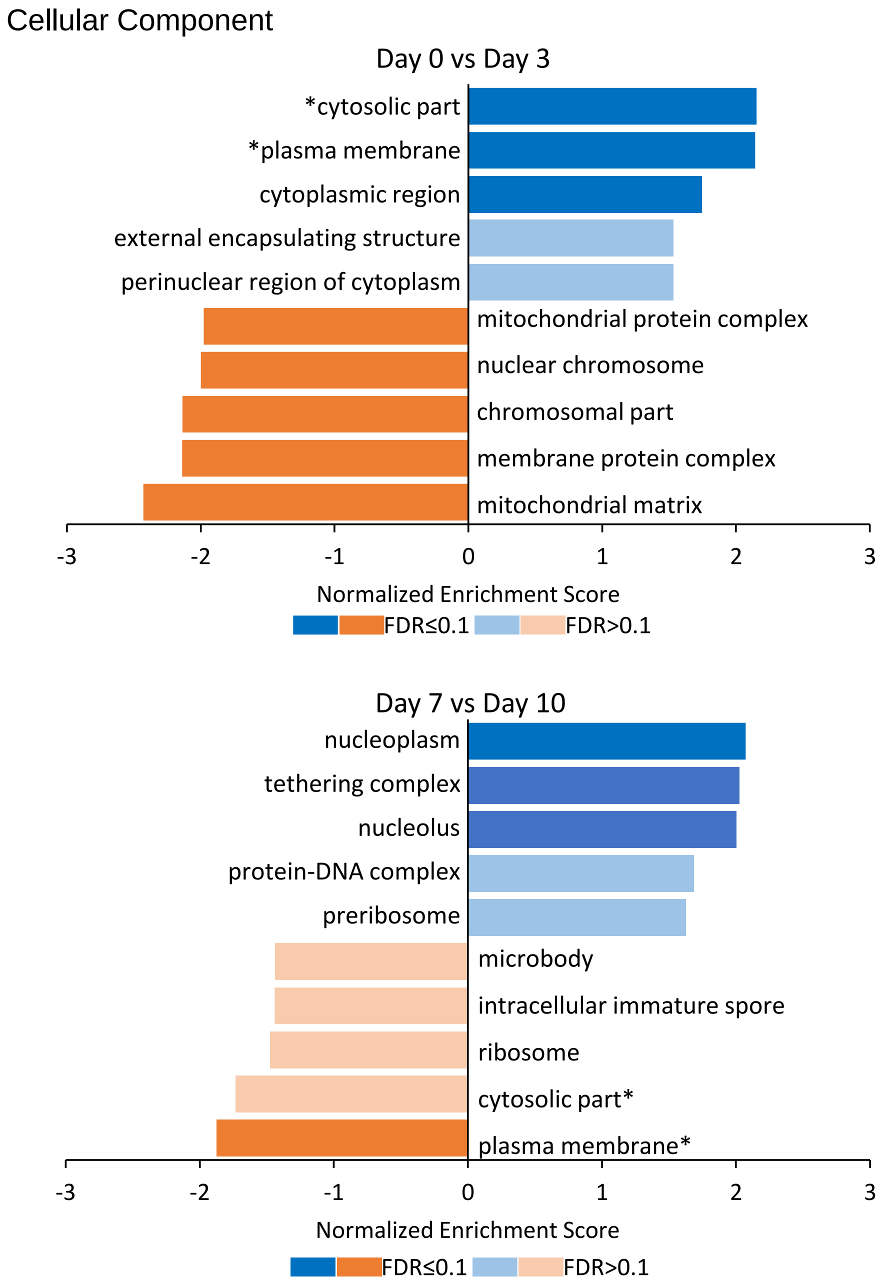

Figure S6. GSEA on differentially compacted loci on the Cellular Component GO domain. Gene Set Enrichment Analysis (GSEA) on the differentially available chromatin loci comparing Day0 vs Day3 and Day7 vs Day10 (the two comparisons displaying most of the significant changes genome-wide). The bar plots show the normalized enrichment score for the top5 gene sets with increased (blue) and decreased (orange) chromatin availability in the latter time point. Dark colors indicate an FDR≤0.1 for the indicated gene set, while pale colors indicate an FDR>0.1. Gene sets that have been identified as increased in the Day0 vs Day3 comparison and decreased in the Day7 vs Day10 comparison (or *vice versa*) are indicated with an asterisk (*).

SUPPLEMENTARY TABLE LEGENDS

Table S1. GSEA summary table. Gene set ID, description, enrichment score and other features of the gene sets identified by the GSEA as a result of consecutive time point comparisons that had a high number of differentially available chromatin regions (Day0 vs Day3 and Day7 vs Day10) for each of the Gene Ontology domains and the KEGG pathways gene set.

Table S2. Oligos used for ATAC-seq library preparation. Primers for ATAC-Seq library preparation. Note that Ad1 is the forward primer, and reverse primers Ad2.1-Ad2.12 carry unique barcodes, highlighted in the sequence in bold italics.

SUPPLEMENTARY FILE LEGENDS

Supplemental File 1. ATAC-seq peaks detected in all samples, temporal dynamics modeling and clustering

Supplemental File 2. Pairwise comparisons of consecutive-timepoint samples obtained with DESeq2.
